# Non-invasive measurement of fasciculation frequency demonstrates diagnostic accuracy in amyotrophic lateral sclerosis

**DOI:** 10.1093/braincomms/fcaa141

**Published:** 2020-09-07

**Authors:** Arina Tamborska, James Bashford, Aidan Wickham, Raquel Iniesta, Urooba Masood, Cristina Cabassi, Domen Planinc, Emma Hodson-Tole, Emmanuel Drakakis, Martyn Boutelle, Kerry Mills, Chris Shaw

**Affiliations:** 1 Department of Basic and Clinical Neuroscience, Maurice Wohl Clinical Neuroscience Institute, King’s College London, London, UK; 2 Department of Bioengineering, Imperial College London, London, UK; 3 Department of Biostatistics and Health Informatics, King’s College London, London, UK; 4 Department of Life Sciences, Musculoskeletal Sciences and Sports Medicine Research Centre, Manchester Metropolitan University, Manchester, UK

**Keywords:** amyotrophic lateral sclerosis, surface electromyography, fasciculation potential, fasciculation frequency, biomarker

## Abstract

Delayed diagnosis of amyotrophic lateral sclerosis prevents early entry into clinical trials at a time when neuroprotective therapies would be most effective. Fasciculations are an early hallmark of amyotrophic lateral sclerosis, preceding muscle weakness and atrophy. To assess the potential diagnostic utility of fasciculations measured by high-density surface electromyography, we carried out 30-min biceps brachii recordings in 39 patients with amyotrophic lateral sclerosis, 7 patients with benign fasciculation syndrome, 1 patient with multifocal motor neuropathy and 17 healthy individuals. We employed the surface potential quantification engine to compute fasciculation frequency, fasciculation amplitude and inter-fasciculation interval. Inter-group comparison was assessed by Welch’s analysis of variance. Logistic regression, receiver operating characteristic curves and decision trees discerned the diagnostic performance of these measures. Fasciculation frequency, median fasciculation amplitude and proportion of inter-fasciculation intervals <100 ms showed significant differences between the groups. In the best-fit regression model, increasing fasciculation frequency and median fasciculation amplitude were independently associated with the diagnosis of amyotrophic lateral sclerosis. Fasciculation frequency was the single best measure predictive of the disease, with an area under the curve of 0.89 (95% confidence interval 0.81–0.98). The cut-off of more than 14 fasciculation potentials per minute achieved 80% sensitivity (95% confidence interval 63–90%) and 96% specificity (95% confidence interval 78–100%). In conclusion, non-invasive measurement of fasciculation frequency at a single time-point reliably distinguished amyotrophic lateral sclerosis from its mimicking conditions and healthy individuals, warranting further research into its diagnostic applications.

## Teaser

Fasciculations are an early hallmark of amyotrophic lateral sclerosis and can be studied noninvasively using high-density surface electromyography. Here we show that fasciculation frequency measured in a 30-minute-long biceps brachii recording differentiates patients with amyotrophic lateral sclerosis from healthy and neurological controls with 80% sensitivity and 96% specificity.

## Graphical Abstract

**Graphical Abstract:**
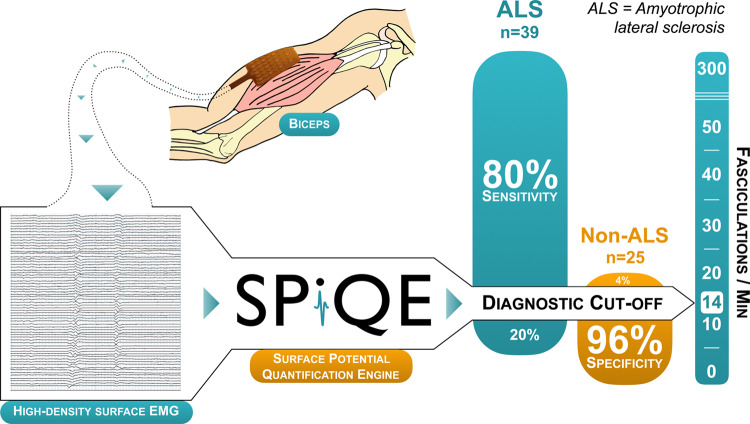

## Introduction

Diagnosis of amyotrophic lateral sclerosis (ALS) is delayed by a median of 12 months after symptom onset (Paganoni *et al.*, 2014), hindering early access to potential neuroprotective treatments and recruitment into randomized controlled trials. The classically insidious onset of ALS means that significant neuronal damage has occurred by the time a diagnosis can be reliably made. It has been estimated that over a third of anterior horn cells have died by the point muscle weakness develops, while widespread electrophysiological changes at the point of diagnosis suggest pre-clinical dissemination of the disease (Swash and Ingram, 1988). Early identification of pre-symptomatic individuals, before irreversible damage occurs, may widen the therapeutic window (Benatar *et al.*, 2019). However, universal and replicable biomarkers of this pre-clinical phase remain to be established (Turner *et al.*, 2009).

Fasciculation potentials (FPs) are an early electrophysiological hallmark of ALS. They can be recorded in clinically strong muscles, where markers of acute denervation, such as fibrillation potentials and positive sharp waves, are absent (de Carvalho and Swash, 1998, 2013). The utility of FPs in the early diagnosis of ALS has been acknowledged in the Awaji criteria (de Carvalho *et al.*, 2008). FPs of greater amplitude and complexity are considered evidence of ongoing denervation of equal importance to fibrillations and positive sharp waves (de Carvalho *et al.*, 2008). In comparison with the El Escorial criteria, the Awaji criteria increased the sensitivity of ALS diagnosis from 62.2% to 81.1% without compromising on specificity (Costa *et al.*, 2012).

High-density surface electromyography (HDSEMG) can detect FPs over large surface areas in strong muscles (Drost *et al.*, 2007). It provides several benefits over traditional needle electromyography (NEMG). First, its non-invasive nature allows for prolonged recording times and serial measurements. Furthermore, whilst NEMG records from a hemisphere of 1 mm radius (Mills, 2005), HDSEMG can cover surface areas greater than 20 cm^2^ (Drost *et al.*, 2007), collecting information from a much larger number of motor units. Finally, the ease of the application and automated analysis reduces expert burden and makes HDSEMG an appealing candidate for ALS monitoring outside of specialist clinical settings (Bashford *et al.*, 2020a).

In this study, we used HDSEMG with a novel, automated quantification engine to investigate the characteristics of FPs in ALS, ALS-mimicking conditions and in healthy individuals. Our aim was to compare the FP measures derived from HDSEMG between the groups and to assess their diagnostic utility.

## Materials and methods

### Study design

This was a retrospective analysis of data from three observational studies at King’s College Hospital (London, UK). Ethical approval was obtained from Research Ethics Services in the North of Scotland (Ref: 15/NS/0103), East Midlands (Ref: 17/EM/0221) and Yorkshire and the Humber (Ref: 19/YH/0164).

### Participants

Eligible patients attending the motor nerve clinic at King’s College Hospital between January 2016 and January 2019 were invited to participate. Females and males between 18 and 80 years of age, who were ambulatory and able to give informed consent were recruited into two groups: ALS (*n* = 39) and neurological controls (*n* = 8).

In the ALS cohort, individuals were deemed eligible if they were diagnosed with possible, probable or definite ALS (according to El Escorial criteria) within 24 months of symptom onset. Individuals with atypical or regionally restricted ALS and individuals with advanced ALS (wheelchair-bound or dependent on non-invasive ventilation) were excluded. Subjects with bulbar onset had to demonstrate objective involvement of at least one limb.

In the neurological control cohort, participants were deemed eligible if they were diagnosed with an ALS-mimicking disorder by a neurologist with an expertise in motor neuron disease. Diagnostic uncertainty was the main exclusion criterion. This group comprised of individuals with benign fasciculation syndrome (BFS) and one individual with multifocal motor neuropathy. Electronic health records of these individuals were retrospectively reviewed at minimum 2 years from the initial recruitment and none of the individuals developed ALS.

Healthy controls (*n* = 17) were recruited from the Manchester Metropolitan University between May and September 2019 via poster adverts. Male and female subjects were deemed eligible if they were between 18 and 80 years of age, were ambulatory, able to provide informed consent and had no self-reported history of any neurological disorder.

### Clinical assessment

Demographic data and the site and the date of first symptoms (where applicable) were recorded for all participants. All participants with ALS or an ALS-mimicking disorder underwent baseline neurological examination, with the ALS subjects classified into phenotypes: bulbar, cervical, lumbar and flail arm. To characterize the disease severity, the ALS subjects had the revised ALS functional rating scale (ALSFRS-R) recorded at the time of the assessment.

### High-density surface EMG recording

Each participant underwent 30-min long HDSEMG recordings from biceps brachii bilaterally or unilaterally depending on the original study protocol. Biceps brachii was selected as one of the large muscles where fasciculations are infrequent in healthy (Fermont *et al.*, 2010) and common in diseased individuals (Suzuki *et al.*, 2018). The adhesive HDSEMG sensor consisted of 64 electrodes (8 × 8 grid, electrode diameter 4.5 mm, inter-electrode distance 8.5 mm; TMS International BV, The Netherlands). Participants were asked to relax on the examination couch in semi-recumbent position with forearms pronated and elbows flexed at 90–120° and the sensor applied centrally over the biceps. A reference electrode (3 × 5 cm) was placed over the ipsilateral olecranon. The signal was amplified using the Refa-64 Recording System (TMS International, BV, Netherlands). The raw data were stored as a Polybench file at a rate 2048 Hz/channel.

### Computational analysis of the HDSEMG data

HDSEMG recordings were processed using surface potential quantification engine (SPiQE): a novel automated analytical tool for the detection and characterization of FPs. We have described the development and validation of SPiQE elsewhere (Bashford *et al.*, 2019). In brief, the first step involves automatic exclusion of perimeter channels and poorly performing channels (such as those with limited signal, baseline drift, artefacts and excessive noise). Next, the remaining channels are merged into a single ‘super-channel’ based on the greatest peak-peak amplitudes for each spike. SPiQE then identifies FPs based on a specific threshold of amplitude. This threshold is automatically adjusted to account for the baseline noise in each recording. Periods of voluntary motor unit activity (i.e. muscle contractions) are excluded using a combination of manual and automatic strategies (Bashford *et al.*, 2020b). SPiQE achieves 88% accuracy in the automated identification of FPs compared with manual counts (Bashford *et al.*, 2019). It provides output on fasciculation frequency, fasciculation amplitude, inter-fasciculation intervals (IFIs) and recording quality (Bashford *et al.*, 2019).

### Development of fasciculation potential measures

Four summary measures were derived from each HDSEMG recording: fasciculation frequency, fasciculation median amplitude, fasciculation amplitude dispersion and proportion of IFIs shorter than 100 ms. Fasciculation frequency describes the number of FPs divided by minutes of the analysed recording. The median amplitude is the median voltage of all FPs detected in each recording. The amplitude interquartile range is the difference between the FP amplitude at the third and the first quartile and, as such, is a measure of amplitude dispersion. IFI describes the interval between two successive FPs. Identical FPs occurring within 100 ms represent fasciculation doublets, indicating axonal hyperexcitability (Mills, 2010). Since SPiQE does not discern FP morphology, proportion of IFI < 100 ms is an experimental measure of axonal hyperexcitability.

### Statistical analysis

Where bilateral biceps recordings were obtained, we calculated their average for each participant. We used descriptive statistics to summarize the characteristics of all groups. We transformed the values to log (10) and assessed the distribution with Q–Q plots and the Shapiro–Wilk normality test. We compared the groups using Welch’s one-way analysis of variance with the Games–Howell *post hoc* test. To investigate the associations of HDSEMG measures with the diagnosis of ALS, we combined neurological and healthy control groups, dichotomized the diagnostic outcome (1 = ALS, 0 = not ALS) and used univariate and multivariate binary logistic regression. To assess the diagnostic performance and identify diagnostic cut-offs, we conducted receiver operating characteristic (ROC) analysis and applied a chi-squared automatic interaction detector (CHAID) decision tree with 3-fold internal validation and a minimum number of eight cases in the parent node and four in the child node. We carried out the analysis using IBM SPSS Statistics software (Version 26, IBM Corp. Armonk, NY, USA). Unless stated otherwise, we rounded the results to the nearest third decimal for *P*-values and nearest second decimal for the remaining data. We set the statistical significance threshold as *P* < 0.05.

### Data availability statement

Deidentified participant data are available upon reasonable request from the corresponding author (J.B.), subject to a CC BY license.

## Results

### Participant characteristics

Thirty-nine patients with ALS, 8 patients with ALS-mimicking conditions and 17 healthy individuals participated in the study (Table 1). Most patients with ALS were male, with definite or probable El-Escorial diagnosis, of spinal phenotype and within 2 years from the symptom onset. The neurological control group consisted of six patients with BFS, one patient with BFS later diagnosed with slowly progressive neuropathy and one patient with multifocal motor neuropathy. All participants completed the planned recording time, yielding over 2,500 minutes of HDSEMG data. Three recordings from ALS patients were excluded from fasciculation analysis due to excessive noise.

**Table 1:** Characteristics of participants included in the study

	ALS (*n* = 39)		Neurological controls (*n* = 8)		Healthy controls (*n* = 17)	
Gender						
Male (%)		30 (77%)		7 (87.5%)		10 (59%)
Female (%)		9 (23%)		1 (12.5%)		7 (41%)
Age						
Median (IQR)		61 (55–66)		39.5 (37.5–55.5)		42 (39–61)
Diagnosis						
El Escorial definite		12 (31%)	BFS	6 (75%)	Healthy	17 (100%)
El Escorial probable		25 (64%)	MMN	1 (12.5%)		
El Escorial possible		2 (5%)	BFS with slowly progressive neuropathy	1 (12.5%)		
ALS phenotype						
Bulbar		10 (26%)	Not applicable		Not applicable
Cervical		17 (43.5%)						
Lumbar		9 (23%)						
Flail arm		2 (5%)						
Cervical/lumbar		1 (2.5%)
Time from symptom onset to assessment						
Median (IQR)		23 months (17.5–32)		24 months (10–26.5)^a^	Not applicable	
ALSFRS-R at the time of assessment						
Median ALSFRS-R (IQR)		42 (38–44)	Not applicable		Not applicable
ALSFRS-R Not recorded (%)		11 (28%)	Not applicable		Not applicable
Bilateral recording analysed						
Yes (%)		24 (61.5%)		7 (87.5%)		4 (23.5%)
No (%)		15 (38.5%)		1 (12.5%)		13 (76.5%)

a Data missing for one participant.

ALS, amyotrophic lateral sclerosis; ALSFRS-R, amyotrophic lateral sclerosis functional rating scale revised; BFS, benign fasciculation syndrome; IQR, interquartile range; MMN, multifocal motor neuropathy.

### Comparison of fasciculation potential measures

**Figure 1:**
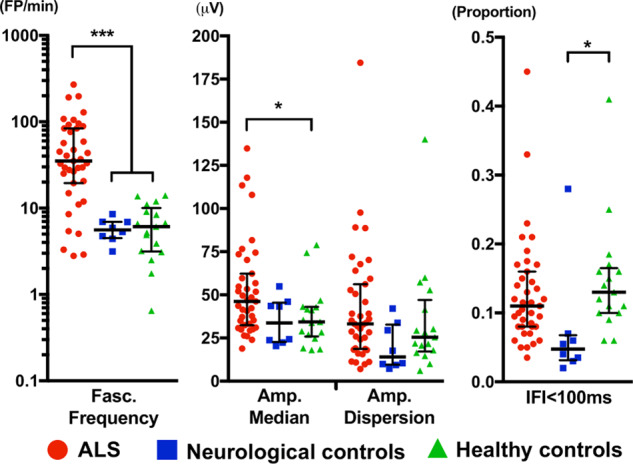
**Comparison of fasciculation potential measures between the groups.** Groups were compared using Welch one-way analysis of variance and Games–Howell *post hoc* test following base 10 logarithmic transformation. Normal distribution of transformed values was confirmed using Q–Q plot inspection and Shapiro–Wilk normality test. Bars across the data points indicate medians with interquartile ranges. Degrees of freedom were approximated to first decimal point, *P*-values to the third decimal point and other results to the second decimal point. ***P < 0.001. *P < 0.05. ALS, amyotrophic lateral sclerosis; Amp., amplitude; Fasc., fasciculation; FP, fasciculation potential; IFI, inter-fasciculation interval.

Comparison of FP measures is summarized in Fig. 1 and Table 2. The most pronounced difference was in the fasciculation frequency, where the count in the ALS group was six times greater than in neurological and healthy controls (*P* < 0.001). Median fasciculation amplitude and amplitude dispersion were also the highest in the ALS patients, but, in the *post hoc* analysis, only the difference in the median fasciculation amplitude between the ALS and the healthy control group was significant (*P* = 0.046). There were trends towards significance in comparison of the median fasciculation amplitude between the ALS and neurological control groups (*P* = 0.091) and amplitude dispersion between ALS and the neurological control group (*P* = 0.06). Proportion of IFIs <100 ms was the highest in the healthy cohort with a significant difference between the healthy and the neurological control groups (*P* = 0.031) and a trend towards significance between the ALS and the neurological controls (*P* = 0.072).

**Table 2:** Comparison of fasciculation potential measures between the groups

	ALS (*n* = 39)	Neurological controls (*n* = 8)	Healthy controls (*n* = 17)	Statistical significance
Fasciculation frequency (FP/min)				
Median (IQR)	35.2 (19.5–84)	5.6 (4.49–6.94)	6.1 (3.15–10.05)	Welch’s *F*_2,33.2_ = 36.81 *P* < 0.001
Fasciculation amplitude (µV)				
Median (IQR)	46.2 (32.5–62.35)	33.73 (22.71–45.43)	34.4 (25.93–43)	Welch’s *F*_2,19.3_ = 4.43 *P* = 0.026
Amplitude dispersion (µV)				
Median (IQR)	33.2 (18.7–56.2)	14 (9.54–32.73)	25.5 (17.2–47.1)	Welch’s *F*_2,18.5_ = 3.35 *P* = 0.058
Proportion of IFIs <100 ms (%)				
Median (IQR)	11 (8–16)	4.75 (3.13–6.75)	13 (10–16.5)	Welch’s *F*_2,18.5_ = 4.54 *P* = 0.027

ALS, amyotrophic lateral sclerosis; FP, fasciculation potential; IFI, inter-fasciculation interval; IQR, interquartile range.

### Association of fasciculation potential measures with the diagnosis of ALS

In the univariate logistic regression, increasing fasciculation frequency [odds ratio (OR) = 1.21, 95% confidence interval (CI) 1.08–1.37, *P* = 0.002] and increasing median amplitude (OR = 1.04, 95% CI 1.01–1.07, *P* = 0.019), but not amplitude dispersion and proportion of IFI <100 ms, were significantly associated with the diagnosis of ALS (Supplementary Table 1). Fasciculation frequency explained the greatest proportion of variance in the diagnosis of ALS (Supplementary Table 1). In the multivariate analysis of all four variables, only increasing fasciculation frequency was independently associated with the diagnosis of ALS (OR = 1.27, 95% CI 1.07–1.52, *P* = 0.007, Supplementary Table 1). Multicollinearity was not present (Supplementary Table 1). The redundancy of the variables was examined using automated backward stepwise selection. The best-fit model consisted of fasciculation frequency, median fasciculation amplitude and proportion of IFIs <100 ms, with both fasciculation frequency (OR = 1.27, 95% CI 1.07–1.52, *P* = 0.008) and median amplitude (OR = 1.07, 95% CI 1.01–1.14, *P* = 0.028) showing a significant positive association with the diagnosis of ALS (Supplementary Table 1). The proportion of variance in the diagnosis of ALS explained by the best-fit three-variable model was comparable to the univariate fasciculation frequency model (Supplementary Table 1).

### Assessment of the diagnostic performance

**Figure 2:**
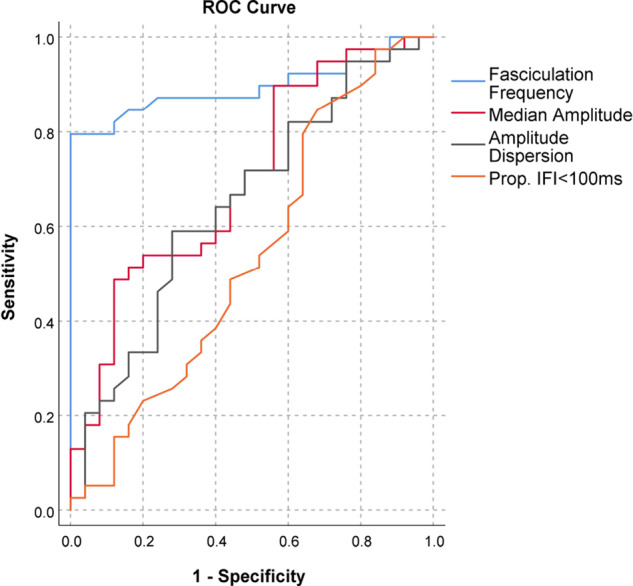
**Assessment of the diagnostic performance of fasciculation potential measures in ALS.** ALS, amyotrophic lateral sclerosis; Prop. IFI <100 ms, proportion of inter-fasciculation intervals <100 ms; ROC, receiver operating characteristic.

We next hypothesized that fasciculation frequency was the single best variable predictive of ALS with limited diagnostic value of other fasciculation measures. To explore this, we first carried out ROC analysis of all four HDSEMG summary measures (Fig. 2). As expected, fasciculation frequency had the best diagnostic performance, with the area under the curve (AUC) of 0.89 (95% CI 0.81–0.98). The remainder of the summary measures did not show diagnostic utility (median amplitude AUC = 0.70, 95% CI 0.57–0.83; amplitude dispersion AUC = 0.65, 95% CI 0.51–0.79; proportion of IFIs <100 ms AUC = 0.54, 95% CI 0.39–0.69).

**Figure 3:**
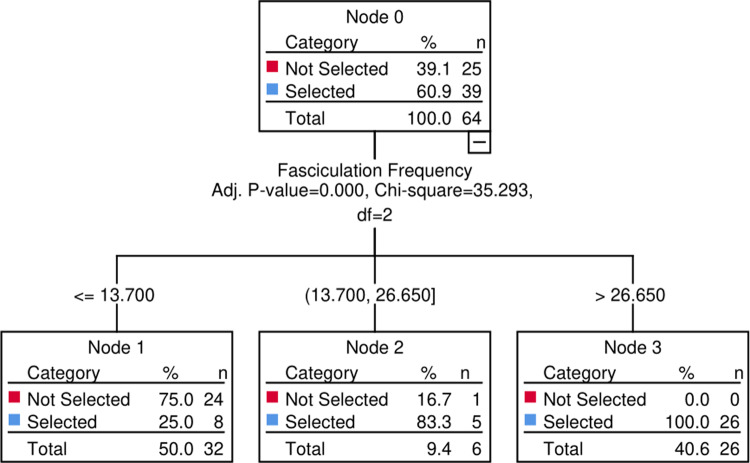
**Decision tree classification model of ALS (Selected—blue) vs. no ALS (Not Selected—red) with 3-fold internal cross-validation.** The model achieved 85.9% correct prediction, confirming fasciculation frequency as a single best diagnostic measure. ALS, amyotrophic lateral sclerosis; CHAID, chi-squared automatic interaction detector; *df*, degrees of freedom.

We then used a CHAID decision tree with 3-fold internal validation (Fig. 3). This confirmed fasciculation frequency as the single best diagnostic measure, delivering 85.9% predictive accuracy (95% CI 75–93.4%). Other summary measures did not offer additional predictive gain. A cross-validated cut-off of 13.7 FPs/minute provided 79.5% sensitivity (95% CI 63.1–90.1%), 96% specificity (95% CI 77.7–99.8%), 96.9% positive predictive value (95% CI 82–99.8%) and 75% negative predictive value (95% CI 56.3–87.9%), confirming the potential for application of fasciculation frequency measured by HDSEMG in the diagnosis of ALS.

## Discussion

Development of universal biomarkers remains a priority in ALS research. The diagnostic value of fasciculation counts has been suggested in ultrasound studies (Tsuji *et al.*, 2017), reporting improved fasciculation detection in addition to standard NEMG (Grimm *et al.*, 2015). However, evidence from HDSEMG studies, which has the advantage of being non-operator dependent, has been limited. In this analysis of over 2,500 minutes of HDSEMG recordings from 64 participants, we show that fasciculation frequency was six times greater in ALS than in healthy and neurological controls. We establish fasciculation frequency as the single best HDSEMG fasciculation measure predictive of ALS, providing 80% sensitivity and 96% specificity. Our results support non-invasive FP quantification by HDSEMG as a promising adjunct in the diagnosis of ALS.

Our neurological control group consisted largely of patients with BFS (87.5%), but previous studies comparing fasciculation frequency in ALS and BFS have yielded contrasting results. A NEMG study of the biceps brachii (Trojaborg and Buchthal, 1965), a multi-muscle NEMG study (de Carvalho and Swash, 1998) and a NEMG study of the first distal interosseous (de Carvalho *et al.*, 2016) reported higher fasciculation rates in BFS than in ALS patients. Conversely, a surface electromyography study of the first dorsal interosseous in patients with BFS and patients with ALS 4–18 months from the diagnosis reported similar fasciculation frequency in both conditions (de Carvalho and Swash, 2016). Matching results were reported by a NEMG study of tibialis anterior of patients with ALS relatively early in their disease course (de Carvalho and Swash, 2013), whereas a multi-muscle NEMG of BFS and ALS patients 18 months from the symptom onset found faster fasciculation firing rates in the ALS subgroup (Mills, 2010).

Such contrasting results could be explained by different disease stages in the ALS subjects. We have recently proposed that fasciculation frequency increases in early ALS, peaking around the time that muscle weakness occurs, after which it subsequently declines (Bashford *et al.*, 2020c). This may account for some authors reporting increasing fasciculation frequency as ALS progresses (Mills, 2010), while others recording lower fasciculation rates in more advanced disease (de Carvalho and Swash, 1998, 2013). Hence, an HDSEMG recording at the peak of the fasciculation frequency curve would lead to more pronounced results. This may explain why we observed a significant difference between ALS and BFS individuals when others did not. Furthermore, it may also explain why eight individuals with ALS had similar fasciculation frequencies to the control groups, which could be due to being relatively early or late in their disease course. Whilst an HDSEMG recording at a single time point differentiated patients with ALS with 85.9% accuracy, patients early in their disease may benefit from serial recordings to enhance diagnostic certainty. Previous ultrasound (Fermont *et al.*, 2010) and NEMG (de Carvalho and Swash, 2013) studies reported few to no FPs in healthy individuals. Our increased pick-up rate, even after accounting for a possible overestimate due to automatic detection, may reflect the longer duration and larger area of HDSEMG recording.

Fasciculation amplitude is a marker of the size of the hyperexcitable motor units. As ALS progresses, the size of the individual motor units increases due to the process of chronic partial denervation (Krarup, 2011). Surviving motor units branch out to supply the orphaned muscle fibres, maintaining muscle power despite the reduced motor unit numbers (Krarup, 2011). We observed significantly greater fasciculation amplitudes in ALS patients than in the control groups, in keeping with the increased motor unit sizes. This is consistent with a NEMG study of tibialis anterior, which also identified greater fasciculation amplitude in individuals with ALS than with BFS (de Carvalho and Swash, 2013). In that study, the amplitude was even greater in individuals with more advanced ALS (de Carvalho and Swash, 2013). However, others found less pronounced differences (Mills, 2010; de Carvalho and Swash, 2016). As with fasciculation frequency, this may be due to different disease stages in ALS patients. Fasciculation amplitude is determined not only by the size of the motor unit, but also by the fasciculation origin in the motor pathway. It is postulated that in more advanced ALS, fasciculations originate distally in the immature branches of the axonal arborization, as opposed to the axonal hillock in early disease (de Carvalho and Swash, 1998). This transition to distal origin may result in partial activation of a motor unit and therefore a reduced fasciculation amplitude. Hence, as ALS progresses, fasciculation amplitude may initially increase due to re-innervation and then decrease again as fasciculation origin moves more distally (Bashford *et al.*, 2020c). This might restrict the diagnostic utility of fasciculation amplitude to subjects in the intermediate stage of disease.

As some motor units increase in size, we expected the amplitude dispersion to be increased in ALS patients. The lack of significant difference may be explained by less frequent fasciculations of the motor units at the extremes of the amplitude. Motor units of normal sizes are less hyperexcitable, whilst very large motor units stop fasciculating and die (Bashford *et al.*, 2020c), capping amplitude dispersion at both ends. Further, because two simultaneous FPs are not discernible by SPiQE, small amplitude FPs from normal sized motor units may have been overridden by large discharges, also limiting the amplitude spread. The proportion of IFI <100 ms in ALS individuals was similar to the proportion of doublets counted manually in weak tibialis anterior muscles in ALS patients (de Carvalho and Swash, 2013). However, these authors saw few doublets in strong ALS patients, no doublets in BFS individuals and no FPs at all in healthy individuals (de Carvalho and Swash, 2013), whilst we recorded the greatest proportion of IFI <100 ms in healthy controls. Whilst prolonged monitoring time and larger recording surface may account for increased FP detection in our healthy controls, it is likely that the experimental cut-off of IFI <100 ms carries a poor correlation with fasciculation doublets. IFIs nearing 100 ms may represent voluntary activity and lower cut-offs may be more specific for truly identical discharges in the absence of morphological analysis.

We aimed to investigate the utility of HDSEMG in differentiating ALS from healthy individuals and neurological mimics. The major strength of our study is the large volume of muscle recordings, with over 2,500 minutes of HDSEMG analysed in an automated manner, removing observer bias. To account for individual variation and symptom asymmetry, we aimed to obtain bilateral recordings, and to improve the assessment of the diagnostic utility, we included both a healthy and a neurological control group. The non-invasive recording was well tolerated by the participants, allowing for a prolonged monitoring time. It was also easy to set-up and analyse, minimizing the expense of technical and electrophysiological expertise.

The main limitation is recruitment of participants with an established diagnosis of ALS at a median of 2 years from the symptom onset. Although this is similar to previous reports, the utility of proposed measures requires external validation in a large prospective study, recruiting participants at the first presentation. We previously stratified patients with ALS by the disease stage (pre-, peri- and post-muscle weakness), showing that fasciculation frequency may be greatest in the intermediate phase of disease (Bashford *et al.*, 2020c). Here, further stratification by the time from the disease onset was limited by the sample size. Furthermore, inclusion of age-matched controls would eliminate the possibility of confounding due to the 20 years age difference between the cohorts in our study. Finally, whilst biceps brachii recording allowed us to observe significant differences, investigation of other muscles at multiple time points may provide additional diagnostic insight.

In conclusion, we show that fasciculation frequency measured by HDSEMG at a single time-point can reliably distinguish ALS from ALS-mimicking conditions and healthy individuals. This finding adds to the growing evidence of the diagnostic utility of HDSEMG in ALS (Zhang *et al.*, 2014; Sleutjes *et al.*, 2015). Together with the non-invasive nature of HDSEMG recording, our findings warrant further investigation into the diagnostic applications of HDSEMG both in specialist and in community settings. Better understanding of the electrophysiological trajectory of ALS will further help to optimize the diagnostic cut-offs, establishing sensitive and specific biomarkers, capable of detecting the disease at an early stage. In a heterogeneous and insidious disease like ALS, it is likely that the ultimate diagnostic approach will require a combination of clinical, electrophysiological, biochemical and imaging biomarkers.

## Supplementary data

Supplementary material is available at *Brain Communications* online.

## Funding

A.T. carried out the work as part of a clinical academic foundation programme. J.B. was supported for 14 months by the Sattaripour Charitable Foundation and the Motor Neurone Disease Association (Shaw/Jul15/932-794). Funding for 36 months was provided through the Medical Research Council/Motor Neurone Disease Association Lady Edith Wolfson Clinical Research Training Fellowship (MR/P000983/1). A.W. was funded by a PhD studentship in the Engineering and Physical Sciences Research Council’s Centre for Doctoral Training in Neurotechnology for Life and Health. U.M., C.C. and D.P. contributed as part of the Clinical Neuroscience MSc course at King’s College London. R.I.’s input represents independent research supported by the NIHR BioResource Centre Maudsley at South London and Maudsley NHS Foundation Trust (SLaM) & Institute of Psychiatry, Psychology and Neuroscience (IoPPN), King’s College London. The views expressed are those of the authors and not necessarily those of the NHS, NIHR, Department of Health or King’s College London.

## Competing interests

The authors report no competing interests.

## Supplementary Material

fcaa141_Supplementary_Data

## Abbreviations

ALS =: amyotrophic lateral sclerosis;
ALSFRS-R =: amyotrophic lateral sclerosis functional rating scale revised;
AUC =: area under the curve;
BFS =: benign fasciculation syndrome;
CHAID =: chi-squared automatic interaction detector;
CI =: confidence interval;
FP =: fasciculation potential;
HDSEMG =: high-density surface electromyography;
IFI =: inter-fasciculation interval;
IQR =: interquartile range;
NEMG =: needle electromyography;
OR =: odds ratio;
ROC =: receiver operating characteristic;
SPiQE =: surface potential quantification engine
