# Supplementary material for: Non-invasive measurement of fasciculation frequency demonstrates diagnostic accuracy in amyotrophic lateral sclerosis
Source: Brain Commun. 2020 Sep 7;2(2):fcaa141. doi: 10.1093/braincomms/fcaa141 (PMC7850269; doi:10.1093/braincomms/fcaa141)
Supplement: fcaa141_Supplementary_Data [file fcaa141_supplementary_data.pdf]

SUPPLEMENTARY TABLE 1

	Univariate Logistic Regression				Multivariate Logistic Regression				Multivariate Logistic Regression of the Best-Fit Model				Collinearity	
**Variable**	**Odds ratio**	**95% CI**	**Sig.**	**Nagelkerke R^2**	**Odds ratio**	**95% CI**	**Sig.**	**Nagelkerke R^2**	**Odds Ratio**	**95% CI**	**Sig.**	**Nagelkerke R^2**	**Tolerance**	**VIF**
**Fasciculation Frequency**	1.21	1.08-1.37	0.002	0.67	1.27	1.07-1.52	0.007	0.73	1.27	1.07 - 1.52	0.008	0.73	0.87	1.15
**Median Amplitude**	1.04	1.01-1.07	0.019	0.16	1.07	1.00-1.14	0.058		1.07	1.01-1.14	0.028		0.32	3.12
**Amplitude Dispersion**	1.02	0.99-1.04	0.159	0.05	1.01	0.97-1.04	0.810		-	-	-		0.43	2.33
**Proportion IFIs <100ms**	2.07	0.004-1046.46	0.819	<0.001	<0.001	<0.001-4.23	0.079		<0.001	<0.001-3.68	0.076		0.64	1.57

Supplementary Table 1. Association of fasciculation potential measures with the diagnosis of ALS.
Best-fit model was identified using automated backward regression. The best-fit model, excluding the amplitude IQR, explained the same proportion of variance in the diagnosis of ALS (indicated by Nagelkerke R^2) as the four-variable model and a similar proportion to the univariate fasciculation frequency model. Collinearity was not present. P values are approximated to the third decimal. Other values are approximated to the second decimal. $P<0.05$ denotes statistical significance.

ALS= Amyotrophic Lateral Sclerosis, IFI= Inter-fasciculation Interval, CI= Confidence Interval, Sig.= Significance, VIF= Variance Inflation Factor.
